# High-Frequency Oscillatory Ventilation With Subcutaneous Drainage in Children With Postintubation Tracheal Injury

**DOI:** 10.1155/crcc/5163174

**Published:** 2025-08-22

**Authors:** Tananat Virojtriratana, Lanlana Nimmankiatkul, Theerapon Jariyasakoolroj

**Affiliations:** Division of Critical Care Medicine, Department of Pediatrics, Faculty of Medicine, Chiang Mai University, Chiang Mai, Thailand

**Keywords:** high-frequency oscillatory ventilation, pneumomediastinum, pneumothorax, subcutaneous drainage, subcutaneous emphysema, tracheal injury

## Abstract

**Introduction:** Tracheal injury during intubation is an uncommon condition in pediatric patients with a high mortality rate. There is no definitive consensus on the management of pediatric postintubation tracheal injury; most studies report satisfactory conservative treatment. High-frequency oscillatory ventilation (HFOV) with bedside subcutaneous drainage using a butterfly needle may be useful for conservative treatment in this group.

**Case Summary:** A 1-year and 7-month-old girl experienced tracheal injury after emergency intubation. The patient developed massive subcutaneous emphysema (MSE), pneumothorax, and pneumomediastinum, accompanied by a worsening clinical condition. The conventional mechanical ventilator (CMV) failed to support this patient; thus, the physician decided to switch to HFOV and used a bedside butterfly needle for subcutaneous air drainage. The patient's condition progressively improved following this procedure.

**Conclusion:** HFOV is beneficial for patients who are failing with CMV since it minimizes air leak and enhances oxygenation and ventilation. Using a butterfly needle for simple subcutaneous drainage at the bedside is more practical and convenient than other techniques. This technique might be advantageous in rapidly improving a patient's condition in a resource-limited setting.

## 1. Introduction

Tracheal injury during intubation is an uncommon condition in pediatric patients, with recorded incidences ranging from 0.03% to 0.12% [1, 2]. Given the mortality risk of up to 75% for pediatric tracheal rupture during intubation [3], early detection and management are crucial to improving patient's outcomes. Despite a successful planned intubation, tracheal injury must be considered in patients presenting with massive subcutaneous emphysema (MSE), pneumothorax, and/or pneumomediastinum following intubation [4], or an unusual degree of air leak relative to mechanical ventilation settings. There is no definitive consensus on the management of pediatric postintubation tracheal injury; most studies report satisfactory conservative treatment [1, 3, 5–7], but some indicate surgical intervention for those who fail conservative treatment [8] or have lesions over 1–2 cm in size [9, 10].

Conventional mechanical ventilation (CMV) using lung-protective strategies is essential for conservative management while awaiting the healing of the trachea. Some patients who do not respond to CMV may benefit from high-frequency oscillatory ventilation (HFOV) [11, 12] while studies are limited. MSE is another condition that affects chest wall compliance and can affect management. Previous literature reviews attempt to evaluate the modalities for managing MSE [13]; nevertheless, certain modalities may be limited in specific centers.

We present a case of a pediatric patient with tracheal injury following intubation due to septic shock. The patient experienced MSE, pneumothorax, and pneumomediastinum in addition to pediatric acute respiratory distress syndrome (PARDS). HFOV was used for rescue management following the failure of CMV, and a butterfly needle was placed into the subcutaneous tissue for bedside drainage.

## 2. Case Summary

**Figure 1:**
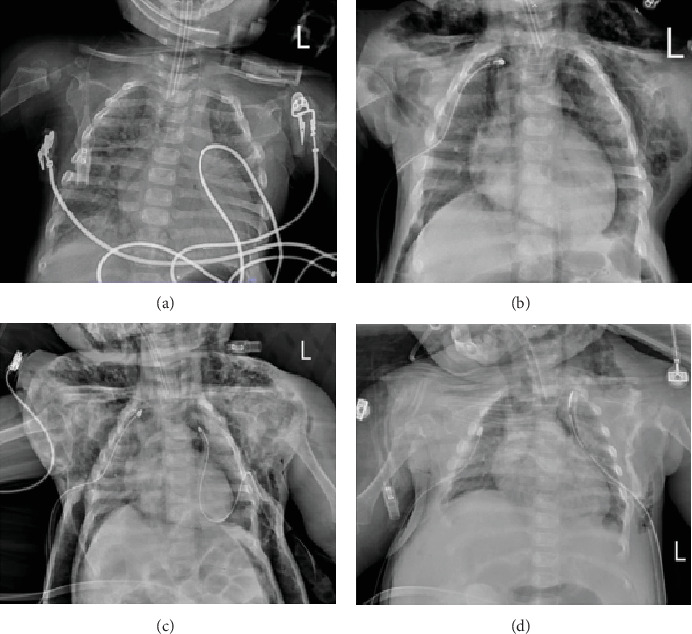
(a) Chest x-ray following intubation at the emergency department. (b) Chest x-ray after to the recognition of subcutaneous emphysema and the insertion of intercostal drainage in the right chest wall. (c) Chest x-ray showing the progression of subcutaneous emphysema during conventional mechanical ventilation mode. (d) Chest x-ray after the use of high-frequency oscillatory ventilation and the insertion of a butterfly needle into the subcutaneous area for air drainage.

A 1-year and 7-month-old girl on peritoneal dialysis (PD) for end-stage renal disease (ESRD) presented with progressive dyspnea and acute fever. The patient's vital signs in the emergency department (ED) were body temperature of 37.1°C, heart rate of 166 bpm, respiratory rate of 60/min, blood pressure that could not be assessed, and oxygen saturation of 86% on room air. The emergency physician successfully performed a 4-mm cuffed endotracheal intubation with a stylet on the first attempt using a gentle technique and without difficulties.  Figure 1a shows the initial chest x-ray. Initially, they inflated the cuffed balloon to around 5 mL without applying cuff pressure. The patient was transferred to the pediatric intensive care unit (PICU). Initial physical examination at the PICU revealed MSE around the chest wall and the neck. A right pneumothorax was identified by bedside ultrasonography. Intercostal drainage (ICD) was immediately inserted, and a chest x-ray was performed, as shown in Figure 1b. The initial CMV setting and blood gas are shown in Table 1. Subsequently, the patient's condition deteriorated, with a lowering blood pressure and expanding MSE to the neck and abdominal areas. MSE, pneumothorax, and pneumomediastinum caused the physician to suspect a large airway injury. With immediate needle thoracotomy, the ICD was inserted on the bilateral side. A computed tomography (CT) scan was done, and the results indicated a potential tracheal injury in the posterior column of the lower trachea. The rupture region was confirmed by the bedside bronchoscope, and it was observed to be approximately 1 cm in length at the posterior part of the lower trachea, with an upper-to-carina distance of approximately 1 cm.

**Figure 2:**
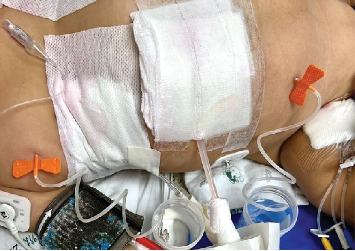
Insertion of a butterfly needle into the subcutaneous area of the chest and abdominal wall.

The MSE was progressing, affecting ventilation, and the blood gas parameters deteriorated (Table 1). The patient's condition deteriorated, and MSE expanded, as shown in Figure 1c. The physician decided to switch ventilator mode to HFOV and used a butterfly needle for bedside drainage of the MSE in the chest and abdominal walls due to being unable to ventilate, using two butterfly needles on each side, resulting in a total of four butterfly needles, as shown in Figure 2. Syringe suction, combined with manual massage, was used every 6 h to facilitate the drainage of accumulated air, resulting in the evacuation of a large volume of air, ranging from 100 to 200 mL. The butterfly needle was drained into the bedside water bottle. Following this, the clinical condition revealed improvement, shown in Table 1 and Figure 1d. The patient underwent HFOV and used a butterfly needle for drainage for a duration of 6 days, resulting in clinical improvement. The physician considered switching from HFOV to CMV; however, the clinical condition deteriorated again within 8 h after reverting, resulting in the development of MSE. The patient had been set for emergency tracheal repair surgery. Upon return from the surgery, the butterfly needle remained inserted subcutaneously to facilitate the release of air. The patient's condition progressively improved, the butterfly needle was removed, and the patient was extubated. The total duration of ventilator use was 28 days.

## 3. Discussion

We describe the successful use of HFOV and a butterfly needle for drainage of MSE from tracheal rupture as initial treatment in a patient undergoing conservative treatment. This patient fails to use CMV due to impaired gas exchange, increased air leak, and clinical deterioration. We used HFOV early, resulting in rapid control of air leaks and improved gas exchange. Previous studies attempted to evaluate the benefits of HFOV in PARDS. HFOV can enhance oxygenation but does not significantly improve hemodynamic stability or mortality outcomes [14, 15]. There have been several reports on the use of HFOV in cases of massive air leakage due to tracheal rupture. Our understanding indicates that HFOV reduces mechanical strain and stress. HFOV ventilation uses minimal tidal volume and high mean airway pressure (Paw) while revealing fewer pressure swings, which is advantageous for lung protection. We aim to use this advantage to ventilate the patient, proposing that a lower pressure swing and minimal tidal volume may decrease the degree of air leak, maintain the patency of the atelectatic region, and enhance both oxygenation and ventilation throughout the period of tracheal healing.

Another concern is MSE in the neck, chest, and abdominal areas, which can impair chest wall compliance and promote inadequate ventilation. A literature study on the treatment of subcutaneous emphysema has been conducted [13], and reports have been made about the effective use of vacuum-assisted closure therapy for MSE in children [16]. This approach has advantages, although it possesses limitations. We use an easy technique with a butterfly needle inserted into the subcutaneous area for air drainage. Considering the limited size of the needle, we use the syringe for negative suction and manual massage every 6 h to augment air drainage, together with bilateral ICD using negative wall suction. The technique successfully reduces subcutaneous air without complications and can potentially be easily performed in resource-limited settings.

We cannot definitively conclude that an overinflated cuff balloon causes tracheal injury in this case; nonetheless, we advocate for monitoring cuff pressure and determining an appropriate volume for inflation. The decision between conservative treatment and surgical intervention must consider the benefits and risks of mortality.

## 4. Conclusion

Tracheal injury after intubation should be considered in patients presenting with MSE, pneumothorax, and/or pneumomediastinum, even in the absence of a history of difficult intubation. The patient for whom conservative treatment is considered may benefit from the use of HFOV to minimize additional air leaks and improve both ventilation and oxygenation. Using a butterfly needle for simple subcutaneous drainage at the bedside is more practical and convenient than other techniques for patients who are unable to ventilate due to MSE. This technique might be advantageous in rapidly improving a patient's condition without complications in resource-limited settings.

## Figures and Tables

**Table 1:** Mechanical ventilator setting and blood gas value.

**Conventional mechanical ventilation (Servo-i)**	

The initial ventilator setting following intubation PC/AC mode: PIP 20, PEEP 8, RR 40, Ti 0.6, FiO_2_ 1.0	VBG: pH = 7.18, PCO_2_ = 41.0, PO_2_ = 45.0 HCO_3_ = 14.6, BD = 13.6, lactate -, OI -
Ventilator setting following an increase in the degree of air leakage PC/AC mode: PIP 24, PEEP 8, RR 50, Ti 0.6, FiO_2_ 1.0	ABG: pH = 7.15, PCO_2_ = 56.0, PO_2_ = 92.0 HCO_3_ = 17.8, BD = 8.7, lactate = 1.2, OI = 16.3

**High-frequency oscillatory ventilation (Sensormedics 3100B)**	

Ventilator settings following the switch to HFOV HFOV mode: *Δ*P 80, Paw 18, F 8, Ti 35%, FiO_2_ 1.0, bias flow 20	ABG: pH = 7.30, PCO_2_ = 45.0, PO_2_ = 119.0 HCO_3_ = 22.1, BD = 4.5, lactate = 1.1, OI = 15.1
Ventilator setting following a decrease in the degree of air leakage HFOV mode: *Δ*P 80, Paw 18, F 9, Ti 35%, FiO_2_ 0.9, bias flow 20	ABG: pH = 7.44, PCO_2_ = 29.0, PO_2_ = 165.0 HCO_3_ = 22.4, BD = 4.5, lactate = 1.7, OI = 9.8

Abbreviations: *Δ*P, delta pressure (amplitude); ABG, arterial blood gas; BD, base deficit; F, frequency; FiO_2_, fraction of inspired oxygen; HFOV, high-frequency oscillatory ventilator; OI, oxygenation index; Paw, mean airway pressure; PC/AC, pressure control/assist control; PEEP, positive end-expiratory pressure; PIP, peak inspiratory pressure; RR, respiratory rate; Ti, inspiratory time; VBG, venous blood gas.

## Data Availability

The data that support the findings of this study are available from the corresponding author upon reasonable request.
